# Neuropsychiatric Outcome of an Adolescent Who Received Deep Brain Stimulation for Tourette's Syndrome

**DOI:** 10.1155/2011/209467

**Published:** 2011-10-19

**Authors:** S. J. Pullen, C. A. Wall, K. H. Lee, S. M. Stead, B. T. Klassen, T. M. Brown

**Affiliations:** ^1^Department of Psychiatry and Psychology, Mayo Clinic, 200 First Street SW, Rochester, MN 55905, USA; ^2^Department of Neurologic Surgery, Mayo Clinic, 200 First Street SW, Rochester, MN 55905, USA; ^3^Division of Child and Adolescent Neurology, Department of Pediatric and Adolescent Medicine, Mayo Clinic, 200 First Street SW, Rochester, MN 55905, USA; ^4^Department of Neurology, Mayo Clinic, 200 First Street SW, Rochester, MN 55905, USA

## Abstract

This case study followed one adolescent patient who underwent bilateral deep brain stimulation of the centromedian parafascicular complex (CM-Pf) for debilitating, treatment refractory Tourette's syndrome for a period of 1.5 years. Neurocognitive testing showed no significant changes between baseline and follow-up assessments. Psychiatric assessment revealed positive outcomes in overall adaptive functioning and reduction in psychotropic medication load in this patient. Furthermore, despite significant baseline psychiatric comorbidity, this patient reported no suicidal ideation following electrode implantation. Deep brain stimulation is increasingly being used in children and adolescents. This case reports on the positive neurologic and neuropsychiatric outcome of an adolescent male with bilateral CM-Pf stimulation.

## 1. Introduction

Tourette syndrome (TS) is a chronic, childhood onset neuropsychiatric disorder, which occurs in approximately one percent of the general population, consists of motor tics and at least one vocal tic lasting longer than one year [1]. Tic symptoms vary in severity, and generally peak between the ages of 8 to 12 years of age, and, in many patients, dissipate by adulthood [2]. Attention deficit hyperactivity disorder (ADHD), obsessive compulsive disorder (OCD), depression, anxiety, and behavioral problems commonly cooccur with TS and compound deficits in psychosocial functioning and overall quality of life [2]. Long-term treatment with antipsychotic medications, adrenergic agonists, and dopamine agonists used alone or in combination with behavioral strategies is often needed in patients with TS and comorbid psychiatric disturbances [3]. 

High-frequency (above 100 Hz) deep brain stimulation (DBS) is being increasingly used as an efficacious treatment modality in patients with severe TS complicated by behavioral and psychiatric comorbidity that is refractory to first and second line treatment [3]. Here, we discuss the neurocognitive and psychosocial outcomes of an adolescent patient who received DBS to treat refractory tics secondary to severe Tourette's syndrome.

## 2. Case Report

A 17-year-old left-handed male with severe Tourette's syndrome, attention deficit hyperactivity disorder (ADHD), and obsessive compulsive disorder (OCD) was evaluated by pediatric neuropsychology and child psychiatry prior to the date of electrode implantation for DBS for treatment of refractory tic symptoms. The patient was treated with bilateral centromedian parafascicular complex (CM-Pf) stimulation and was followed for 1.5 years. Preoperative neuropsychometric testing was noteworthy for borderline general intellectual functioning and commensurate academic achievement (Table 1). Cognitive weaknesses were noted in executive functioning (e.g., abstract reasoning, planning, and organization) and in learning, but memory retention was largely intact. The patient also exhibited bilateral fine motor dexterity impairment and difficulty with visual-motor integration. The patient's father revealed clinically significant concerns about anxiety, depression, somatic behaviors, and atypical behaviors (e.g., repetitive behaviors). He also indicated concerns about hyperactivity, attentional problems, poor adaptability, and poor independent functioning. As per history provided by the patient and his family, the genesis of the patient's psychiatric symptoms coincided with the evolution of the patient's TS. These progressively worsened over time and had been refractory to pharmacologic and nonpharmacologic intervention. The patient presented to our tertiary care center for consideration to initiate DBS treatment due to the severity of his neurologic and neuropsychiatric symptoms. 

Preoperative psychiatric evaluation demonstrated severe psychosocial impairment related to treatment refractory Tourette's syndrome. Social impairment was associated with significant amounts of missed school and anxiety related to tics. Attempts to garner employment were hampered with being overwhelmed by his neurologic symptoms. Past psychiatric history was significant for three prior psychiatric hospitalizations, all occurring within the context of managing a complicated psychotropic medication regimen, and treatment approach for comorbid psychiatric conditions (Table 1). Inpatient hospitalization prior to electrode implantation was recommended to safely taper some of the psychotropic medications that could interfere with DBS treatment. 

Postoperative neuropsychometric testing revealed general cognitive functioning that had remained stable across time (Table 1). The patient continued to have difficulty with learning information but retained information once learned. No significant changes were noted in the patient's cognitive ability compared with baseline testing. 

Postoperative psychiatric evaluation was noteworthy for significant functional and psychosocial gains. No concerns for suicidal or self-injurious thoughts or actions were noted. The patient's psychotropic medication load was also significantly reduced (Table 1). Improved psychosocial functioning corresponded with improvement in tic symptoms (Table 1). 

## 3. Discussion

This case report focused on the neurocognitive and psychosocial outcomes in an adolescent male who had received DBS for severe refractory Tourette's syndrome followed for 1.5 years. This patient demonstrated no significant changes in his follow-up psychometric testing and reported significant improvement in psychosocial functioning postoperatively. He was able to decrease his psychotropic medication load during follow-up psychiatric assessments and did not report any suicidal ideation throughout the course of treatment. 

DBS is being increasingly used for treatment-refractory TS in adults and is an important emerging treatment modality of treatment-refractory TS within the pediatric population [4]. Thus far three different regions of the brain are used for DBS: the CM-Pf and ventralis oralis nuclei complex which are part of the intralaminar nuclei of the thalamus; the anterior and posterior GPi; the anterior portion of the internal capsule of the nucleus accumbens [3]. The CM-Pf is one of the most common sites for DBS stimulation, and bilateral stimulation of the CM-Pf has been shown to be effective in treating both tic and behavioral symptoms of TS, although most of the patients thus far described have been adults [5]. 

Case reports documenting long-term treatment outcomes in both adults and adolescents with severe refractory TS using DBS have yielded mixed results with regards to improved psychosocial functioning and overall quality of life. Stimulation of the globus pallidus internus (GPi) was shown to be beneficial in one adolescent boy with severe TS and comorbid OCD and anxiety but had little therapeutic benefit in another patient with similar tic severity, but lacking comorbid psychiatric conditions [6, 7]. Ackermans et al. also reported variable treatment outcome in two adult patients who had undergone thalamic stimulation for refractory TS [8]. Long-term outcomes in one patient showed improvement in tic severity and overall quality of life, without cognitive sequela; while their other patient showed some regression of initial tic improvement, a decrease in verbal fluency and learning, and continued to show a high level of psychopathology when assessed over a period of six years [8]. 

In a larger prospective cohort study, Porta and colleagues documented improvement in tic symptoms, as well as improvement in neuropsychiatric symptoms without cognitive decline in a 24-month followup of 15/18 patients who underwent bilateral thalamic stimulation [5]. This study predominately evaluated adult patients (mean age of patients in this study being 30 years with a standard deviation of 8.7 years) and was significant in demonstrating the efficacy of DBS in treating severe refractory TS, improving psychiatric sequelae, without any demonstrable neurocognitive deficits over a two-year period [5]. As the use of DBS in adolescents grows, such studies will also be important in this population as well. 

Given the ongoing need for awareness of cognitive and psychiatric outcomes in adolescent patients who have undergone DBS treatment, we specifically focused on the neurocognitive and psychosocial outcomes in an adolescent male who had received DBS for severe refractory TS [9]. This case highlights the positive neuropsychiatric outcome for an adolescent patient who had received DBS with bilateral centromedian parafascicular complex stimulation for severe Tourette's syndrome and adds to the small but growing data describing treatment outcomes of adolescent patients who have underwent DBS for treatment refractory TS. Larger studies assessing neurologic, neurocognitive, and neuropsychiatric treatment outcomes in young people undergoing DBS for neuropsychiatric treatment are needed.

## Figures and Tables

**Table 1:** Results of neurologic examination, neurocognitive testing, and psychotropic medication load pre- and postelectrode implantation.

Electrode placement	Bilateral centromedian parafascicular complex (CM-Pf)	Preimplantation	Postimplantation
Neurologic testing	YGTSS Scores^a^	Date of testing 12-22-2009	Date of testing 08-05-2010

	Number motor	2	1
	Number phonic	1	0
	Frequency motor	5	1
	Frequency phonic	2	0
	Intensity motor	5	2
	Intensity phonic	2	0
	Complexity motor	2	0
	Complexity phonic	1	0
	Interference motor	5	0
	Interference phonic	2	0
	Overall impairment	50	10
	Global severity	77	14

Neurocognitive testing	WAIS-III/IV^b^	Date of testing 12-23-2009	Date of testing 08-02-2010

	Full scale IQ	76	74
	Verbal comprehension	84	78
	Perceptual organization	76	73
	Working memory	80	86
	Processing speed	76	81

Psychosocial testing	BASC^c^	Date of testing 12-23-2009	Date of testing 08-02-2010

*Clinical scales*	Hyperactivity	68	59
	Aggression	52	45
	Conduct problems	56	56
	Anxiety	79	59
	Depression	78	52
	Somatization	69	54
	Atypicality	69	49
	Withdrawal	53	42
	Attention problems	63	55

*Adaptive scales*	Adaptability	33	49
	Social skills	52	62
	Leadership	40	53
	Activities daily living	32	45
	Functional communication	39	53

*Composite scales*	Externalizing problems	60	54
	Internalizing problems	81	56
	Behavioral symptoms index	68	50
	Adaptive skills	37	53

Psychiatric evaluation	Psychotropic medications^d^	Date of evaluation 12-22-2009	Date of evaluation 08-05-2010

		Aripiprazole 30 mg Diazepam 20 mg Divalproex sodium 3000 mg Guanfacine 2.5 mg Quetiapine 400 mg Risperidone 1.5 mg	Risperidone 6 mg Guanfacine 2 mg

^a^YGTSS:Yale Global Tic Severity Scale.

^b^WAIS-III/IV: Wechsler Adult Intelligence Scale-III/IV—Standard scores with a general population mean of 100 and a standard deviation of 15 are reported here. The WAIS III was administered preimplantation. The WAIS IV was administered postimplantation.

^c^BASC: Behavior Assessment System for Children, Second Edition Ages 12–21; raw test scores reported here. A score reduction on items within *clinical scales* reflects relative improvement in the different measures of psychiatric symptomatology, whereas a score increase on items within *adaptive scales* reflects a relative improvement on different measures of adaptive functioning. Within the *composite scales*, a score reduction in the domains of externalizing symptoms, internalizing symptoms, and behavioral symptoms reflects relative improvement, whereas a score increase in the adaptive skills domain reflects relative improvement.

^d^Psychotropic medications reported as maximum daily dosages prescribed to patient.
